# Daily Vinegar Ingestion Improves Depression Scores and Alters the Metabolome in Healthy Adults: A Randomized Controlled Trial

**DOI:** 10.3390/nu13114020

**Published:** 2021-11-11

**Authors:** Carol S. Johnston, Paniz Jasbi, Yan Jin, Shayna Bauer, Susanna Williams, Samantha N. Fessler, Haiwei Gu

**Affiliations:** College of Health Solutions, Arizona State University, Phoenix, AZ 85004, USA; pjasbi@asu.edu (P.J.); kimyeon909@gmail.com (Y.J.); shaynadbauer@gmail.com (S.B.); williamssuzy98@gmail.com (S.W.); sfessler@asu.edu (S.N.F.); hgu@fiu.edu (H.G.)

**Keywords:** depression, metabolomics, vinegar

## Abstract

Daily vinegar ingestion has been linked to improved glycemic control, but recent data suggest a separate unexplored role for vinegar in mental health. Utilizing a placebo-controlled, parallel arm study design, this 4-week trial examined the impact of daily vinegar ingestion on mood states and urinary metabolites in healthy college students. Participants were randomized to the vinegar group (VIN: *n* = 14; 1.5 g acetic acid/day as liquid vinegar) or the control group (CON: *n* = 11; 0.015 g acetic acid/day as a pill) with no change to customary diet or physical activity. At baseline and at study week four, participants completed the Profile of Mood States (POMS) and the Center for Epidemiological Studies-Depression (CES-D) questionnaires and provided a first-morning urine sample for targeted metabolomics analyses. The change in both POMS depression scores and CES-D scores differed significantly between groups favoring improved affect in the VIN versus CON participants after four weeks. Metabolomics analyses pre and post-intervention suggested metabolite alterations associated with vinegar ingestion that are consistent for improved mood, including enzymatic dysfunction in the hexosamine pathway as well as significant increases in glycine, serine, and threonine metabolism. These data warrant continued investigation of vinegar as a possible agent to improve mood state.

## 1. Introduction

Vinegar, a product of fermented grains and fruit, is emerging as a potential adjunct treatment for several chronic conditions. Daily vinegar ingestion has been linked with reduced postprandial glycemia and hemoglobin A1c concentrations in individuals with type 2 diabetes [1,2,3] and lowered blood triglycerides in obese adults [1,4,5]. While the main component of all vinegars is acetic acid (4–7% *w*/*w*), other compounds and minerals specific to the fermented food source are present in trace amounts [6]. The improved glycemic response is attributed to acetic acid, which has been demonstrated to reduce the digestive capacity of disaccharidases in vitro [7] and to enhance the clearance of glucose from the blood stream into muscle in vivo [4,8]. The lipid lowering mechanism of vinegar may relate to the upregulation of PPAR-α and its target genes acyl-CoA oxidase 1 and carnitine palmitoyltransferase I, and enhanced β-oxidation in the liver [9,10]. Possibly, bioactives in vinegar in concert with acetic acid initiate these effects [9,10].

To explore more comprehensively the biological processes altered by vinegar ingestion, we recently conducted metabolomics analyses in healthy adults consuming red wine vinegar twice daily for eight weeks [11]. Plasma concentrations of two metabolites, amino valerate and indole-3-acetic acid, increased significantly over time in the vinegar group relative to controls, with high magnitudes of fold change (>2) between groups. Metabolic pathway analysis revealed significant alterations in tryptophan metabolism in the vinegar group relative to controls. In rats, metabolomics analysis of urine also identified a change in tryptophan metabolism in the vinegar treated animals in comparison to controls [12]. These data are intriguing given the antioxidant role of indole-3-acetic acid in biological systems, including brain, and the well-documented role of tryptophan in production of the stress-alleviating neurotransmitter serotonin [13,14,15]. These data suggest a potential, unexplored role for vinegar in mental health. Theoretically, vinegar may improve mental function by providing an exogenous source of acetate, a metabolite linked to improved cognitive performance in rats [16]. Also, a diet high in fiber increased acetate levels in vivo via gut fermentation and improved depressive symptoms [17].

Utilizing a placebo-controlled, randomized, parallel arm study design, this research examined the impact of daily vinegar ingestion on mood states and urinary metabolites in healthy adults. Specifically, we tested whether apple cider vinegar ingestion (2 tablespoons twice daily with meals for four weeks) reduced depression scores, based on validated measures of depression, in comparison to the control treatment. Intergroup change in urinary metabolites over time were compared using targeted metabolomics methodology.

## 2. Materials and Methods

### 2.1. Reagents

Acetonitrile (ACN), methanol (MeOH), ammonium acetate (NH_4_OAc), and acetic acid (AcOH), all analytical grade, were purchased from Fisher Scientific (Pittsburgh, PA, USA). Ammonium hydroxide (NH_4_OH) was bought from Sigma-Aldrich (Saint Louis, MO, USA). Deionized water was provided in-house by a Water Purification System from EMD Millipore (Billerica, MA, USA). Phosphate buffered saline (PBS) was bought from GE Healthcare Life Sciences (Logan, UT, USA). Standard compounds corresponding to the measured metabolites were purchased from Sigma-Aldrich (Saint Louis, MO, USA) and Fisher Scientific (Pittsburgh, PA, USA).

### 2.2. Participants

Healthy, normal to underactive (<300 min of moderate physical activity per week and not training for or competing in athletic events) college students (aged 18–23 year) who were free of acute illnesses or chronic diseases (by self-report) were recruited from a campus population using online advertisements in October 2020. Exclusion criteria included pregnancy or lactation, dietary restrictions (e.g., adherence to vegetarian diets, weight loss diets, or medical diets), obesity (BMI ≥ 30 kg/m^2^) or concerns about consuming vinegar daily for four weeks. Due to restrictions imposed by the COVID-19 pandemic, completion of study questionnaires occurred online, and study investigators met participants outdoors to collect urine samples. The Arizona State University Institutional Review Board approved this study (STUDY 00012406), and all participants provided written consent. The study is registered at clinicaltrials.gov (NCT04706806).

### 2.3. Study Protocol

The study followed a placebo-controlled, randomized, parallel arm study design. Participants were stratified by age, gender, and body mass index and randomly assigned by coin toss to a treatment arm. Participants in the active study arm ingested two tablespoons of vinegar (VIN: 5% acidity; Braggs^®^, Liquid Apple Cider Vinegar) diluted in one cup water twice daily with meals for four weeks, and participants in the control arm consumed one vinegar tablet (CON: General Nutrition Corporation Apple Cider Vinegar tablets) daily for four weeks. The liquid vinegar treatment provided 1.5 g acetic acid daily in comparison to the vinegar tablet that provided 0.015 g acetic acid daily. An active dose of acetic acid ranges from 0.7 to 2.0 g daily based on previous research examining the medicinal effects of vinegar; commercial vinegar pills have been used in past studies as a control treatment [11,18]. The vinegar pills had an acidic/vinegar odor and were described to participants as ‘vinegar pills’; hence, participants were blinded to treatment assignment. Participant compliance to the treatment protocol was recorded daily on a paper calendar that was returned to study investigators at the completion of the study.

A spot urine sample from the first morning void was collected at baseline and at study week four. Participants were instructed to collect a midstream sample in the provided sterile container and to tightly reseal the container and place in a paper bag. At baseline, participants met with investigators at an agreed upon outside location the same day urine was collected to turn over the urine sample and receive their four-week supply of liquid or tablet vinegar supplements based on group assignment. This process was repeated at study week four when participants provided urine samples and compliance calendars to the investigators. Additionally, at baseline and at study week four, two validated, widely applied mood questionnaires were completed online: the Profile of Mood States (POMS) and the Center for Epidemiological Studies-Depression (CES-D) questionnaires.

### 2.4. Questionnaires

The POMS questionnaire utilizes 65 adjectives to assess six mood states (tension, depression, anger, fatigue, confusion and vigor) over the past week. Participants responded to each adjective on a 5-point Likert scale ranging from ‘Not at all’ to ‘Extremely’, and scoring was computed for individual mood states and total mood state based on manual instructions with higher scores indicating greater mood disturbance [19]. Normative scores for college students are 44.8 for females and 41.1 for males [19]. The CES-D is a 20-item measure that asks respondents to rate on a four-point scale (0 = Rarely or None of the Time, 1 = Some or Little of the Time, 2 = Moderately or Much of the time, 3 = Most or Almost All the Time) how often over the past week they experienced symptoms associated with depression. Scores range from 0 to 60, with higher scores indicating greater depressive symptoms; a score greater than 15 may indicate an elevated risk of depression [20]. Scores ≥ 30 indicate elevated depressive symptoms that may impact an individual’s health [21].

### 2.5. Urine Metabolomics Analyses

Urinary metabolites and metabolic pathways were analyzed using targeted metabolomics methodology. Data were derived from integrated liquid chromatography-tandem mass spectrometry (LC-MS/MS) peak area values (semi-quantitative). Frozen urine samples were first thawed overnight under 4 °C. Afterward, 50 μL of each urine sample was placed in a separate vial. The initial step for protein precipitation and metabolite extraction was performed by adding 500 μL MeOH and 50 μL internal standard solution (containing 1810.5 μM ^13^C_3_-lactate and 142 μM ^13^C_5_-glutamic acid). The mixture was then vortexed for 10 s and stored at −20 °C for 30 min, followed by centrifugation at 14,000 rpm for 10 min at 4 °C. The supernatants (500 μL) were collected into new Eppendorf vials and dried using a CentriVap Concentrator (Labconco, Fort Scott, KS, USA). The dried samples were reconstituted in 150 μL of 40% PBS/60% ACN and centrifuged again at 14,000 rpm at 4 °C for 10 min. After that, 100 μL of supernatant was collected from each sample into a LC vial for subsequent analysis. A pooled sample was analyzed alongside experimental samples as a quality control (QC) and injected once every 10 experimental samples.

The targeted LC-MS/MS method used here was modeled after that developed and used in a growing number of studies [11,22,23,24]. Briefly, all LC-MS/MS experiments were performed on an Agilent 1290 UPLC-6490 QQQ-MS system. Each supernatant sample was injected twice, 10 µL for analysis using negative ionization mode and 4 µL for analysis using positive ionization mode. Both chromatographic separations were performed in hydrophilic interaction chromatography mode on a Waters XBridge BEH Amide column (150 × 2.1 mm, 2.5 µM particle size, Waters Corporation, Milford, MA, USA). The flow rate was 0.3 mL/min, auto-sampler temperature was kept at 4 °C, and the column compartment was set to 40 °C. The mobile phase was composed of Solvents A (10 mm NH_4_OAc, 10 mm NH_4_OH in 95% H_2_O/5% ACN) and B (10 mm NH_4_OAc, 10 mm NH_4_OH in 95% ACN/5% H_2_O). After an initial 1 min isocratic elution of 90% B, the percentage of B decreased to 40% at t = 11 min. The composition of B was maintained at 40% for 4 min (t = 15 min), after which the percentage of B gradually went back to 90%, to prepare for the next injection. The mass spectrometer was equipped with an electrospray ionization source. Targeted data acquisition was performed in multiple-reaction-monitoring (MRM) mode. The whole LC-MS system was controlled by Agilent MassHunter Workstation software. The extracted MRM peaks were integrated using Agilent MassHunter Quantitative Data Analysis software.

### 2.6. Statistical Analysis

Demographic data, absolute mood scores, and the 4-week change in mood scores (post/pre) are displayed as the mean ± SD. Mann-Whitney and chi-squared analyses were used to assess differences between groups. Spearman’s rank correlation coefficient was used to assess relationships between variables. Based on high-fiber and probiotic feeding trials, marked elevations in serum acetate concentrations do not appear to impact creatinine concentrations in vivo [25,26]; hence, urine metabolite data were normalized to urinary creatinine concentration [27]. Furthermore, creatinine was not significantly affected by vinegar in the current study (*p* > 0.05, independent samples *t*-test). The four-week change data were derived by normalizing treatment values by baseline values (post/pre). Generalized logarithm transformation was applied, and data were Pareto scaled (mean-centered and divided by the square root of the standard deviation of each variable). Univariate analyses were used to pinpoint significant changes in metabolites between groups [11]. Fold change (FC) analysis and orthogonal partial least squares-discriminant analysis (OPLS-DA) were performed to identify the differences in metabolic profiles between groups over time and to operationalize candidate markers for classification, respectively. IBM SPSS Statistics were used for the non-parametric and univariate analyses (IBM Corp. Released 2020. IBM SPSS Statistics for Windows, Version 27.0. Armonk, NY, USA: IBM Corp). Significance was set at *p* < 0.05. Multivariate statistical analyses were performed using open-source R software. Pathway and integrating enzyme enrichment analysis were performed and visualized using MetaboAnalyst 5.0 software package [28]; significance and impact were calculated using a global test of relative between-ness centrality. Results were synthesized and visualized using BioRender.com.

## 3. Results

**Figure 1:**
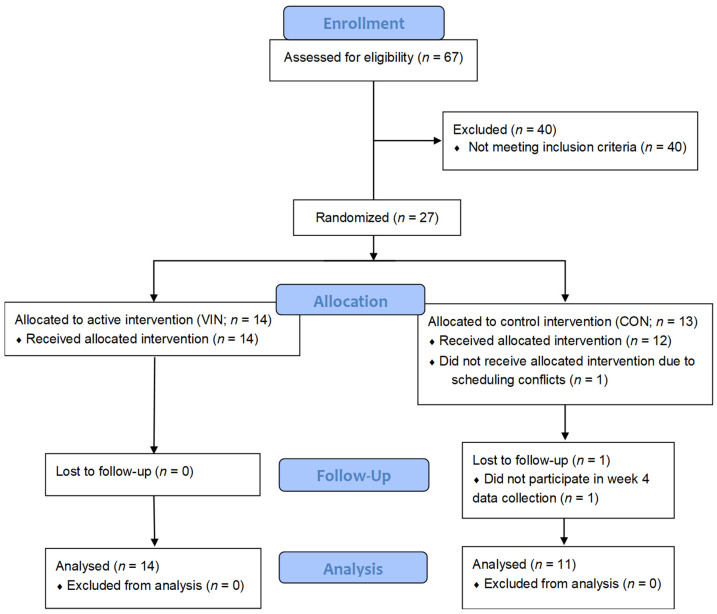
CONSORT flow diagram of study participants.

67 students responded to the online recruitment survey and 27 (40%) met the study criteria and were enrolled in the study (Figure 1). One participant randomized to the control treatment withdrew prior to study initiation due to scheduling conflicts, and a second control participant did not complete week four data collection. Hence, 25 participants completed the four-week study in its entirety, and these data are reported herein (14 VIN and 11 CON). Based on the written calendar record, daily supplement compliance for the VIN and CON groups was 92% and 95% respectively. Participant characteristics did not vary significantly between groups (Table 1); however, there was a 7% difference in age and BMI between VIN and CON participants (0.05 < *p* < 0.10).

### 3.1. Psychological Data

Based on the CES-D scoring at baseline, five VIN participants (36%) and four CON participants (36%) were at an elevated risk for depression. Baseline scores averaged 12.9 ± 6.4 and 13.1 ± 6.9 for the groups respectively (*p* > 0.05; Table 2). At study week four, CES-D scores averaged 8.5 ± 6.8 and 16.2 ± 8.2 respectively for the VIN and CON groups, and the change from baseline was significant between groups (−4.4 ± 4.0 and +3.1 ± 6.9, *p* = 0.002). At week four, four VIN participants and six CON participants scored >15 indicating an elevated risk for depression. None of the scoring at baseline or week four exceeded 30, an indication of elevated depressive symptoms, with the exception of a single CON participant at week 4. At baseline, the total POMS scores averaged 67.8 ± 5.4 and 68.9 ± 5.4 for the VIN and CON groups respectively (*p* > 0.05; Table 2); only one participant in each group scored below the published normative scores for female and male college students (44.8 and 41.1 respectively; see ref. [17]). At study week four, the total POMS scores averaged 49.8 ± 4.3 and 75.6 ± 10.4 for the VIN and CON groups respectively, and the change scores trended differently between groups (−18.0 ± 4.2 and +6.6 ± 11.5 respectively; *p* = 0.085). The four-week change scores for individual mood states did differ significantly for depression (−5.1 ± 1.2 and +2.8 ± 2.6 for the VIN and CON groups; *p* = 0.006) and approached significance for vigor (+2.4 ± 1.0 and −1.3 ± 2.9 respectively; *p* = 0.051). The change in CES-D and POMS depression scores were directly correlated (*r* = 0.479, *p* = 0.015).

### 3.2. Metabolomics Data

**Figure 2:**
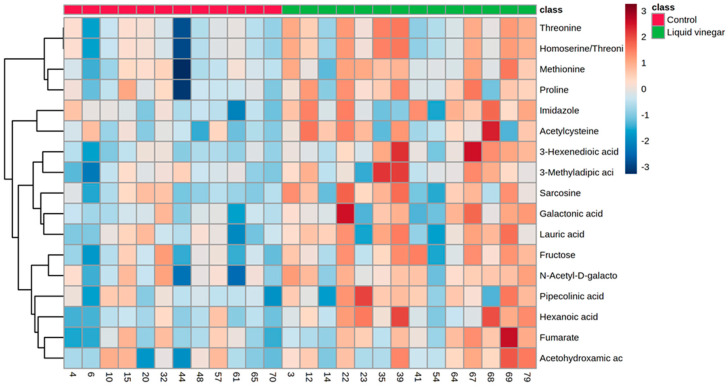
Heat map of 17 metabolites that were identified as significantly altered by independent samples *t*-testing. Data were analyzed as relative change (post/pre) between VIN and CON groups.

**Figure 3:**
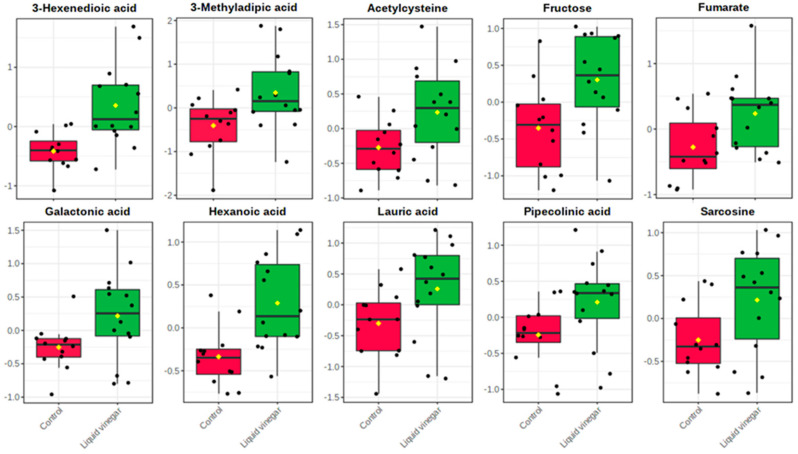
Boxplots of 10 significant metabolites (*p* < 0.05) with large magnitude of change (FC > 2; VIN/CON): 3-hexenedioic acid (0.002), 3-methyladipic acid (0.021), acetylcysteine (0.031), fructose (0.012), fumarate (0.027), galactonic acid (0.041), hexanoic acid (0.002), lauric acid (0.049), pipecolinic acid (0.044), sarcosine (0.038).

Metabolite levels were compared between VIN and CON groups as relative change values (post/pre); 17 metabolites were found to be significant (Supplementary Materials Table S1). Figure 2 displays a heat map for the 17 metabolites that were identified to be significant by independent samples *t*-testing (all relative change values *p* < 0.05). A volcano plot constructed with all reliably detected metabolites, showed 10 of the 17 significant between-group metabolites to be significantly upregulated in the liquid vinegar group with a large magnitude of change (Supplementary Materials Figure S1; FC > 2.0, *p* < 0.05). Specifically, the liquid vinegar group excreted higher levels of metabolites involved in amino acid metabolism (sarcosine, pipecolinic acid, and acetylcysteine) and fatty acid metabolism (3-hexenedioic acid, lauric acid, and hexanoic acid) (Figure 3). In addition, VIN individuals excreted greater concentrations of fumarate (citric acid cycle) as well as galactonic acid and fructose (carbohydrate metabolism) relative to CON individuals (Figure 3).

**Figure 4:**
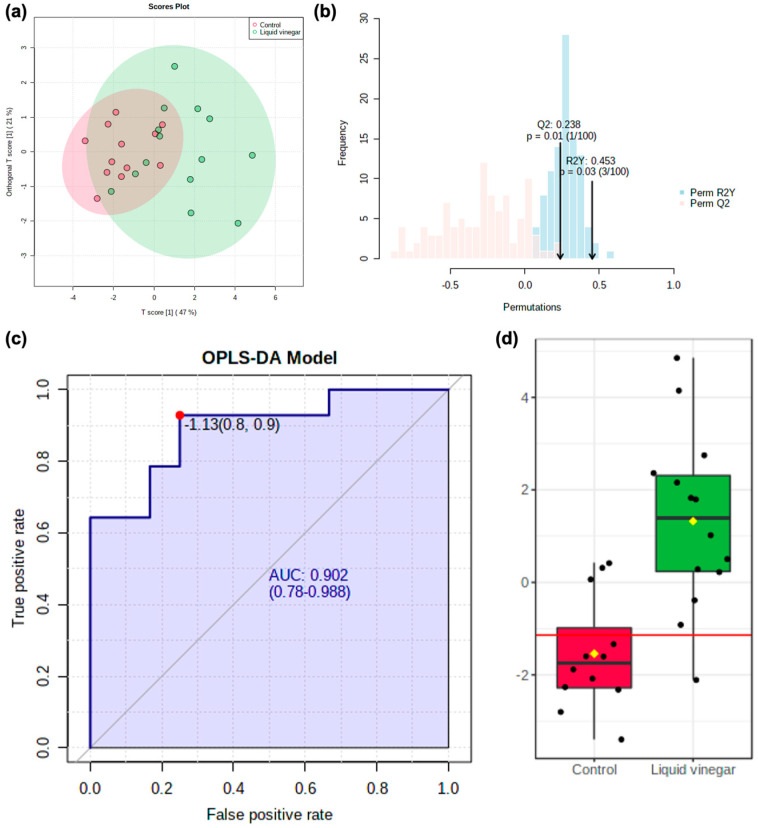
(**a**) Scores plot of OPLS-DA model constructed using 10 significant metabolites (*p* < 0.05) with FC > 2 and univariate AUC > 0.70: *Q*^2^ = 0.238, *R*^2^*Y* = 0.453. (**b**) Permutation testing of OPLS-DA model using 100 iterations (*Q*^2^ *p* = 0.01, *R*^2^*Y* *p* = 0.03). (**c**) ROC curve of the combined 10 metabolite OPLS-DA model for discrimination between control and liquid vinegar groups (AUC = 0.902, 95% CI = 0.780–0.988, sensitivity = 0.90, specificity = 0.80). (**d**) Box plot of OPLS-DA model-implied *Y*-values between groups as derived from OPLS-DA.

Receiver operating characteristic (ROC) analysis was performed on this subset of 10 candidate markers, revealing them all to have an area under curve (AUC) = 0.727–0.857 (Supplementary Materials Figure S2). An OPLS-DA model was then used to operationalize the 10 candidate markers for group classification (Figure 4a). An overview of the model showed modest predictive capacity (*Q*^2^ = 0.238) and appreciable explanatory capacity (*R*^2^*Y* = 0.453) as well as acceptable model fit as determined by 100-fold permutation testing (*p* ≤ 0.03) (Figure 4b). Multivariate ROC analysis of model performance showed excellent overall classification (AUC = 0.902) with high sensitivity (0.9) and robust specificity (0.8) (Figure 4c). Notably, OPLS-DA model-implied *Y*-values were more predictive of study group than any of the 10 candidate metabolites individually (Figure 4d).

**Figure 5:**
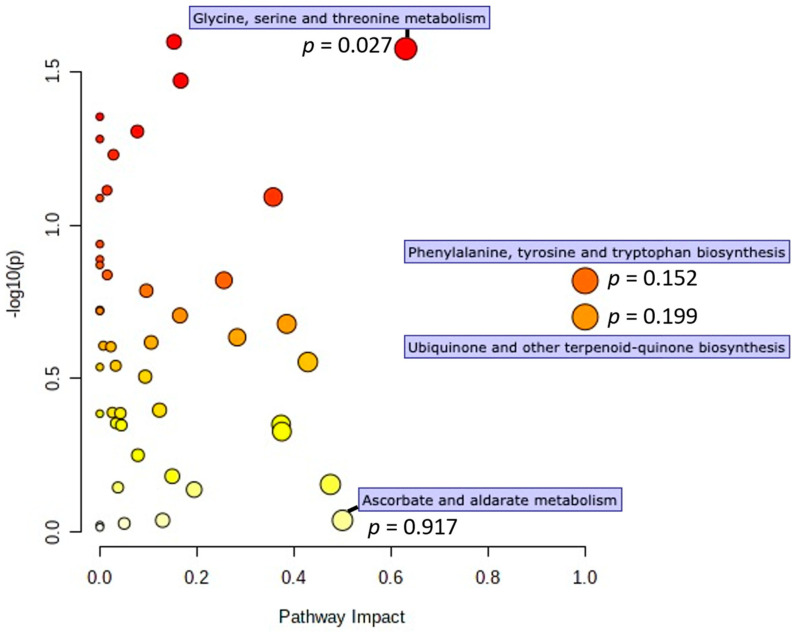
Pathway enrichment analysis performed using all reliably detected metabolites mapped to canonical KEGG pathways. Significance is shown as a function of impact. All pathways with high impact have been marked (>0.50) and associated *p* values are provided.

Observed metabolite change values were mapped to the Kyoto Encyclopedia of Genes and Genomes (KEGG) database and pathway enrichment analysis was performed (Figure 5). Significance and impact were calculated using a global test of relative-betweenness centrality. Four metabolic pathways showed high impact (≥0.50): ascorbate and aldarate metabolism, glycine, serine and threonine metabolism, phenylalanine, tyrosine, and tryptophan biosynthesis, and ubiquinone and other terpenoidquinone biosynthesis. Of these, glycine, serine and threonine metabolism was significantly enriched between study groups (*p* = 0.027). All metabolites in this significantly enriched pathway were elevated in the VIN group.

**Figure 6:**
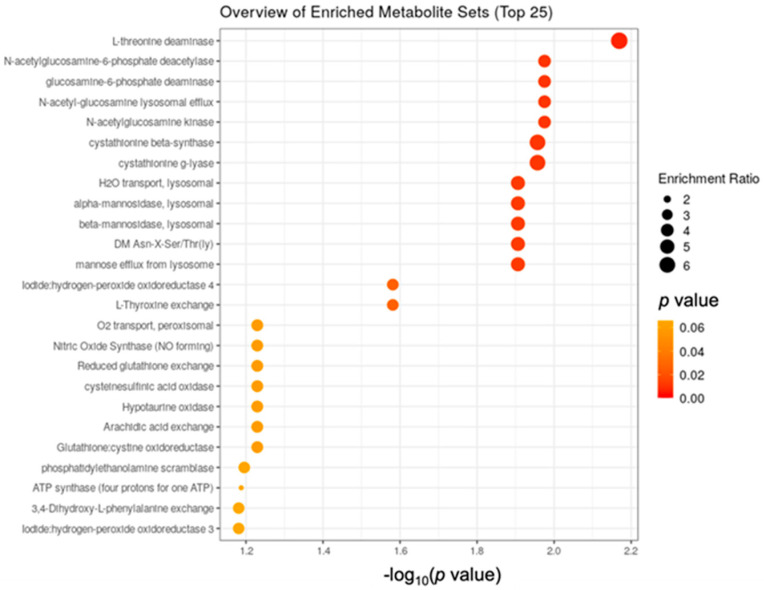
Enzyme enrichment analysis performed using 912 metabolite sets predicted to change in the case of dysfunctional enzymes.

Using library searches of more than 900 metabolite sets predicted to be changed in the case of dysfunctional enzymes, enrichment analysis using least absolute shrinkage and selection operator (LASSO) regression identified 14 enzyme shifts that differed significantly between groups during the four-week study (*p* < 0.05; Figure 6 and Supplementary Materials Table S2). Results are displayed in Figure 6 as a function of enrichment ratio and *p*-value. Enrichment of all 14 significant enzymes was greater in the VIN group. Notably, five of these 14 enzymes were lysosomal in origin.

## 4. Discussion

To our knowledge, this is the first study to link daily vinegar ingestion in healthy young adults with improved mood. Specifically, we noted a 20–34% reduction in self-reported depression scores in participants ingesting vinegar daily for four weeks whereas slight increases in self-reported depression were noted in the control participants over the same interval (*p* ≤ 0.006). The metabolomics data showed clear distinctions between groups during the trial, and we utilized computational methods to integrate the data. Several of the metabolic alterations associated with vinegar ingestion were consistent for improved mood, including enzymatic dysfunction in the hexosamine pathway as well as significant increases in glycine, serine, and threonine metabolism. Acetic acid is the major ingredient in vinegar, and vinegar ingestion results in a rapid rise in serum acetate [29]. Hence, we focused on acetate metabolism as we reviewed and interpreted the biological processes that varied between the VIN and CON groups.

Acetate influences tissue metabolism mainly via the acetyl-CoA signaling pathway. The metabolism of glucose and fatty acids contributes to the acetyl-CoA pool under fed and fasted conditions respectively, and much research has detailed these pathways see [30]. However, acetate from exogenous sources (vinegar ingestion or fiber fermentation in the gut) also contributes to the acetyl-CoA pool, and the acetyl-CoA signaling pathways resulting from exogenous acetate are not well described in humans. A recent report by Zheng et al., demonstrated that the depletion of acetate-producing bacteria in the gut microbiota in a rat model of type 1 diabetes accelerated cognitive decline, and this decline was reversed by oral acetate supplementation [16]. Xie et al., fed a high-cellulose diet to rats and observed an increase in the gut microbiota Bacteroidetes/Firmicutes ratio and a resulting 2-fold increase in acetate. The high fiber diet also increased the concentration of brain derived neurotrophic factor (BDNF), a key molecule involved in plastic changes related to learning and memory [31]. Interestingly, BDNF concentration in the gut was elevated following oral acetate administration to rats treated with an antibiotic cocktail to eliminate gut microbes, suggesting a direct link between acetate and the production of BDNF [31].

Results of the enzyme enrichment analysis reported herein suggest alterations in the hexosamine pathway in adults ingesting liquid vinegar (1.5 g acetic acid daily) compared to control adults ingesting a control tablet (0.015 g acetic acid daily). This pathway is a minor branch of glucose metabolism, and is the source of key substrates for protein glycosylation [32]. Acetyl-CoA enters the hexosamine pathway to participate in the acetylation of glucosamine-6-phosphate [33]. Three enzymes involved in the reversal of this pathway were elevated in the liquid vinegar group compared to controls, *N*-acetylglucosamine-6-phosphate deacetylase (*p* = 0.011), glucosamine-6-phosphate deaminase (*p* = 0.011), and *N*-acetylglucosamine kinase (*p* = 0.011), suggesting that the ingestion of acetic acid attenuates or reverses protein glycosylation. Further evidence of this possibility was the identification of *N*-acetyl-glucosamine and mannose lysosomal efflux pathways and the elevation of lysosomal mannosidases from the enrichment analyses, all indicating degradation of glycosylated proteins. Mannose is an endogenously derived constituent of *N*-glycan structures, and its release from cells is a sign of catabolism of these structures [34].

Research has linked increased flux through the hexosamine biosynthetic pathway to insulin resistance in cultured adipocytes [35]. The hyperglycemia characteristic of the prediabetic and diabetic conditions stimulates the hexosamine pathway due to high tissue uptake of glucose. Although unexplored, this is a possible mechanism for the favorable effects of liquid vinegar ingestion on insulin sensitivity noted in human trials. Moreover, and pertinent to the present trial, the brain contains some of the highest levels of *N*-acetylglucosamine glycated proteins in the body, with the hippocampus expressing high levels of *N*-acetylglucosamine transferase and *N*-acetylglucosaminase [36]. An acute increase in *N*-acetylglucosamine glycosylation can affect neuronal communication by inducing long-term depression of excitatory transmission at hippocampal synapses, as well as suppressing hyperexcitable circuits in vivo [36,37]. In addition, *N*-acetylglucosamine glycosylation reduces action potential probability in pyramidal cells [36]. These findings from previous trials may help explain the improvements in depressive symptoms noted in the present trial.

The pathway enrichment analysis demonstrated a significant increase in glycine, serine and threonine metabolism in adults consuming liquid vinegar in comparison to controls. Serine, a non-essential amino acid, is synthesized from 3-phosphoglycerate, an intermediate in glycolysis. As outlined, the metabolomics data suggests that acetic acid ingestion reverses the hexosamine pathway, and elevations in the metabolite fructose further confirms this. Increased fructose availability may stimulate the glycolysis pathway, which is the source of substrate for serine synthesis. In turn, serine is the most significant precursor to glycine. Both serine and glycine impact neurological health [38,39]. Serine has a role in the synthesis of sphingolipids and glycolipids which are important myelin constituents [40]. Serine also enhances dendritogenesis and axon lengths [41]. Glycine is a neurotransmitter with trophic effects on neurons [38]. Glycine is a substrate in the synthesis of sarcosine, another metabolite increased in the vinegar group in comparison to controls.

**Figure 7:**
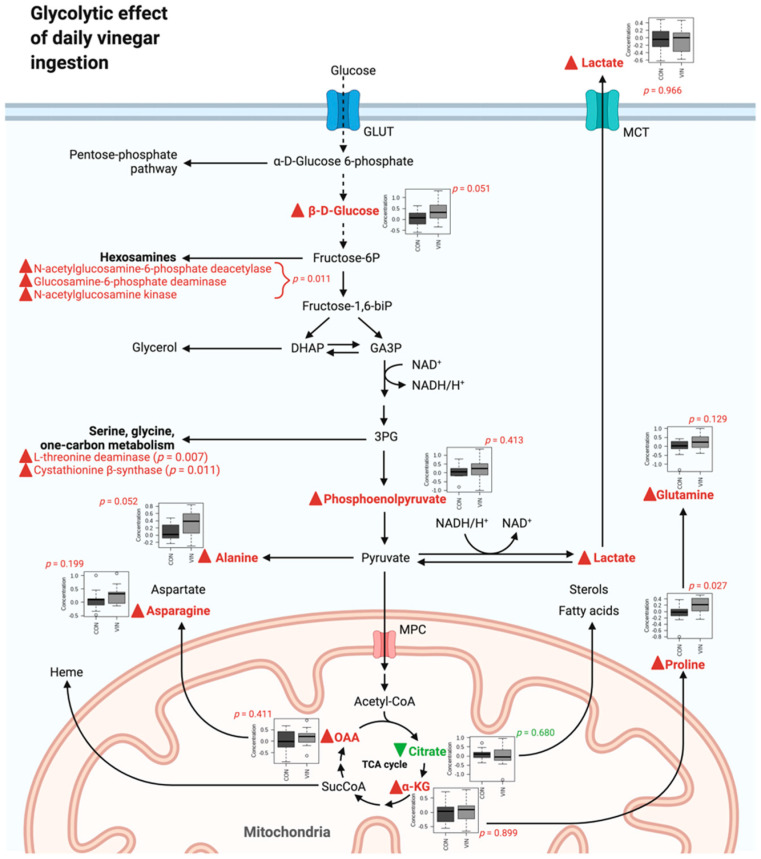
Integrated hypothesis describing observed and predicted metabolic response to vinegar supplementation. The conceptual schema was constructed using results of univariate significance testing, pathway enrichment analysis, and enzyme enrichment analysis. Green lettering with downward green arrows denote decreases in the VIN group, while red lettering and upward red arrows denote increases in the VIN group. Created with BioRender.com.

Our results were synthesized to form an integrated hypothesis describing the effects of vinegar ingestion. Our findings are visualized in Figure 7 within the context of glycolysis, the TCA cycle, and parallel pathways. In the current study, 10 metabolites were reliably detected within these pathways (0.027 < *p* < 0.966). Notably, nine of these metabolites were increased in the VIN group (β-D-glucose, phosphoenolpyruvate, lactate, alanine, α-ketoglutarate, proline, glutamine, oxaloacetate, and asparagine), while only one metabolite in these pathways was decreased in the VIN group (citrate). Our predicted functional results were also incorporated and showed enzymatic dysfunction in the related hexosamine pathway (*p* = 0.011) as well as glycine, serine, and threonine metabolism (0.007 < *p* < 0.011). Box plots and significance information are included in Figure 7 where appropriate.

The metabolomics results described herein provide insights into possible impacts of exogenous acetic acid on metabolism; however, we did not observe alterations in tryptophan metabolism as noted in our previous trial [11]. The previous trial utilized red wine vinegar while the present trial utilized an apple vinegar. The phytochemical profiles of these two vinegars are different; hence, it is possible that phytochemicals in these vinegars influenced metabolism independent of acetic acid. For example, the polyphenol profiles of these vinegars differ with red wine vinegars rich in gallic acid, caffeic acid, and p-coumaric acid, while apple vinegars are rich in protocatechuic acid and chlorogenic acid [42,43,44]. In addition, red wine vinegars are rich in tartaric acid whereas apple vinegars are rich in malic acid [42]. The antioxidant activities of red wine vinegars are 2-fold greater than that of apple vinegar, a characteristic that has been attributed to their phenol content. Antioxidant compounds may protect tryptophan from catabolism and increase tryptophan content in brain [45]. Furthermore, the polyphenols in red wine have been linked to altered protein digestion and tryptophan metabolism in the small intestine [46,47].

The small sample size (*n* = 25) of the present study is a limitation; however, the 95% confidence intervals for the change in depression scores by group do not overlap lending confidence in the results (vinegar: −7.4 to −1.4; control: −0.3 to 6.5). A post-hoc power analysis using the actual change in depression scores reported herein indicated the study was adequately powered (average power = 86%). The moderate correlation between the change in CES-D and POMS-depression scores (*r* = 0.479) across the sample provides added confidence in results. However, these data should be cautiously considered as affect scores were measured at two time points only: pre- and post-intervention. The study was conducted in late October/early November 2020 when U.S. COVID-19 caseloads spiked to the highest point-to-date in the pandemic, and the university campus was closed. The student participants’ depression scores were slightly elevated above normal levels, which may have factored into the results.

In conclusion, these data suggest that daily apple cider vinegar ingestion (2 tablespoons; 5% acidity) lowered depression scores in healthy college students under controlled conditions after four weeks. Metabolomics analyses pre- and post-intervention suggest metabolite alterations associated with vinegar ingestion that are consistent for improved mood. With over 40% of college students self-reporting moderate-to-severe depression, a 77% increase over the past decade [48], simple and safe strategies that effectively reduce depression in this population are urgently needed. These data warrant continued investigation of vinegar as a possible agent to improve mood state.

## Figures and Tables

**Table 1:** Demographics at baseline for the liquid vinegar (VIN) and vinegar pill (CON) groups ^1^.

	VIN	CON	*p*
Age, year	21.00 ± 2.18	19.55 ± 1.37	0.051
Female/Male	10/4	7/4	1.000
Height, in	65.96 ± 4.48	67.82 ± 3.06	0.183
Weight, lbs.	150.07 ± 23.92	147.55 ± 44.82	0.403
BMI, kg/m^2^	24.17 ± 2.41	22.53 ± 6.59	0.066

^1^ Data are the mean ± SD; *p*-value represents Mann Whitney test for ratio data and chi-square test for nominal data.

**Table 2:** Center for Epidemiological Studies-Depression (CES-D) and Profile of Mood States (POMS) scores at baseline (pre) and after the four-week treatment period (post) *.

		Pre	Post	Change	*p*
CES-D	VIN	12.9 ± 6.4	8.5 ± 6.8	−4.4 ± 4.0	0.002
	CON	13.1 ± 6.9	16.2 ± 8.2	3.1 ± 6.9	
POMS	VIN	67.8 ± 5.4	49.8 ± 4.3	−18.0 ± 4.2	0.085
	CON	68.9 ± 5.4	75.6 ± 10.4	6.6 ± 11.5	
Tension	VIN	18.4 ± 1.0	13.6 ± 1.0	−4.8 ± 1.2	0.134
	CON	18.6 ± 1.8	19.4 ± 2.4	0.8 ± 2.4	
Anger	VIN	18.6 ± 1.0	16.6 ± 1.0	−2.0 ± 1.1	0.291
	CON	16.9±1.2	18.2 ± 1.9	1.3 ± 2.4	
Fatigue	VIN	16.0 ± 1.2	13.0 ± 1.2	−3.0 ± 0.7	0.134
	CON	15.9 ± 1.3	15.9 ± 1.9	0.0 ± 1.6	
Depression	VIN	25.2 ± 1.9	20.1 ± 1.1	−5.1 ± 1.2	0.006
	CON	23.7 ± 2.3	26.6 ± 3.0	2.8 ± 2.6	
Vigor	VIN	23.3 ± 1.2	25.6 ± 1.2	2.4 ± 1.0	0.051
	CON	20.9 ± 1.9	19.6 ± 1.7	−1.3 ± 2.9	
Confusion	VIN	12.8 ± 1.0	12.1 ± 0.9	−0.7 ± 0.8	0.403
	CON	14.7 ± 1.3	15.2 ± 1.4	0.5 ± 1.6	

* Data are the mean ± SD; change data calculated as the post-score minus pre-score; *p* represents Mann Whitney test for change data; CES-D and POMS scores do not differ by treatment group at baseline (*p* > 0.05).

## Data Availability

The data presented in this study are available on request from the corresponding author.

## Supplementary Materials

The following are available online at https://www.mdpi.com/article/10.3390/nu13114020/s1, Table S1. Metabolite changes over the trial period that differed significantly between groups as determined by independent samples *t*-test, Table S2. Results of enzyme enrichment analysis, Figure S1. Volcano plot displaying the 10 metabolites to be significantly upregulated (*p* < 0.05) with a large magnitude of change (FC > 2.0) in the liquid vinegar group in comparison to controls, Figure S2. Results of univariate ROC analysis.
