# Supplementary material for: Daily Vinegar Ingestion Improves Depression Scores and Alters the Metabolome in Healthy Adults: A Randomized Controlled Trial
Source: Nutrients. 2021 Nov 11;13(11):4020. doi: 10.3390/nu13114020 (PMC8622118; doi:10.3390/nu13114020)
Supplement: Supplementary file 1 [file nutrients-13-04020-s001.zip › nutrients-1433293-supplementary.pdf]

SUPPLEMENTARY MATERIALS

Daily Vinegar Ingestion Improves Depression Scores and Alters the Metabolome in Healthy Adults: A Randomized Controlled Trial

Carol S Johnston[1*], Paniz Jasbi[1], Yan Jin[1], Shayna Bauer[1], Susanna Williams[1], Samantha Fessler[1], and Haiwei Gu[1]

[1]College of Health Solutions, Arizona State University, Arizona USA; carol.johnston@asu.edu (CSJ); pjasbi@asu.edu (PJ); kimyeon909@gmail.com (YJ); sdbauer2@asu.edu (SB); williamssuzy98@gmail.com (SW); sfessler@asu.edu (SF); hgu@fiu.edu (HG)
[*]Correspondence: carol.johnston@asu.edu

Table S1. Metabolite changes over the trial period that differed significantly between groups as determined by independent samples *t*-test*.

Metabolite	*p*
3-Hexenedioic acid	0.002
Hexanoic acid	0.002
Homoserine	0.007
Threonine	0.007
Methionine	0.011
Fructose	0.012
3-Methyladipic acid	0.021
Imidazole	0.023
N-Acetyl-D-galactosamine	0.026
Fumarate	0.027
Proline	0.027
Acetylcysteine	0.031
Sarcosine	0.038
Galactonic acid	0.041
Acetohydroxamic acid	0.043
Pipecolinic acid	0.044
Lauric acid	0.049

*Significance calculated using change values (post/pre) between VIN and CON groups.

Table S2. Results of enzyme enrichment analysis.

**Enzyme**	**Total**	**Hits**	**Statistic**	***p***
L-threonine deaminase	3	1	26.783	0.007
N-acetylglucosamine-6-phosphate deacetylase	5	3	16.009	0.011
glucosamine-6-phosphate deaminase	6	3	16.009	0.011
N-acetyl-glucosamine lysosomal efflux	8	3	16.009	0.011
N-acetylglucosamine kinase	8	3	16.009	0.011
cystathionine beta-synthase	6	1	24.014	0.011
cystathionine g-lyase	6	1	24.014	0.011
H_2O transport, lysosomal	5	2	19.373	0.012
alpha-mannosidase, lysosomal	7	2	19.373	0.012
beta-mannosidase, lysosomal	7	2	19.373	0.012
DM Asn-X-Ser/Thr(ly)	7	2	19.373	0.012
mannose efflux from lysosome	7	2	19.373	0.012
Iodide: hydrogen-peroxide oxidoreductase 4	25	2	13.786	0.026
L-Thyroxine exchange	25	2	13.786	0.026

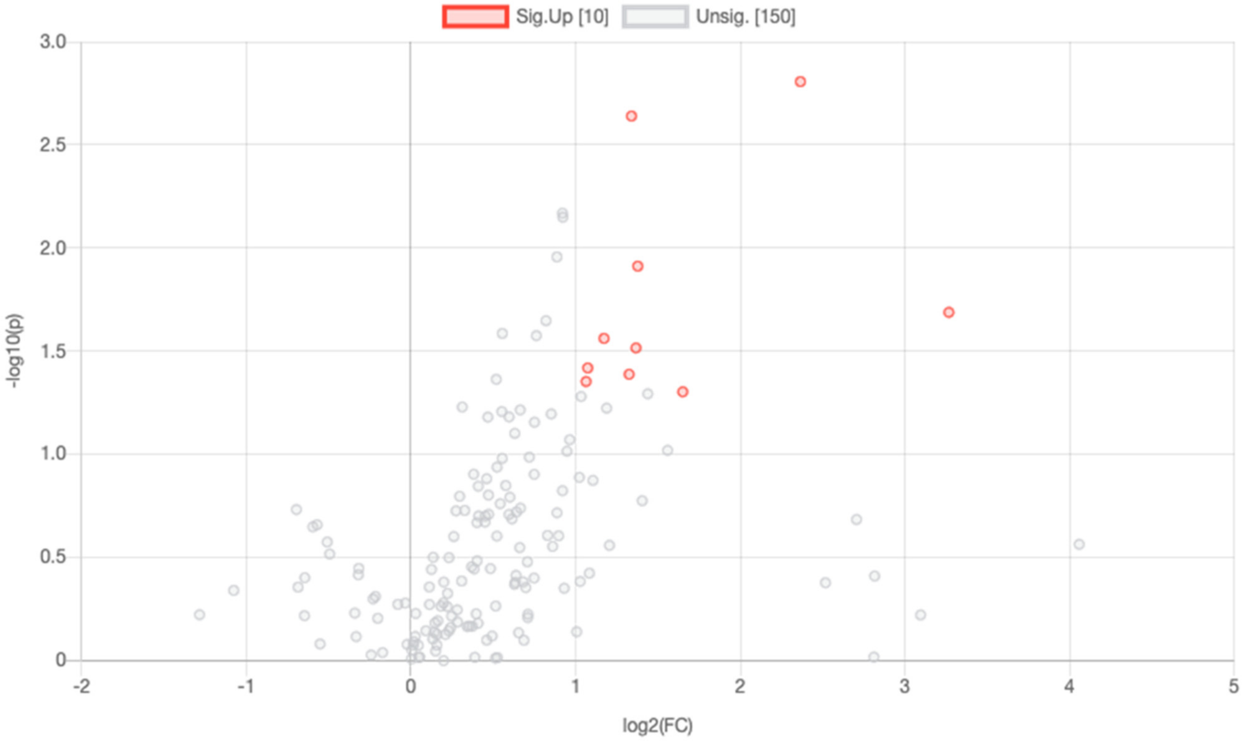

Figure S1. Volcano plot displaying the 10 metabolites to be significantly upregulated ($p < 0.05$) with a large magnitude of change (FC > 2.0) in the liquid vinegar group in comparison to controls.

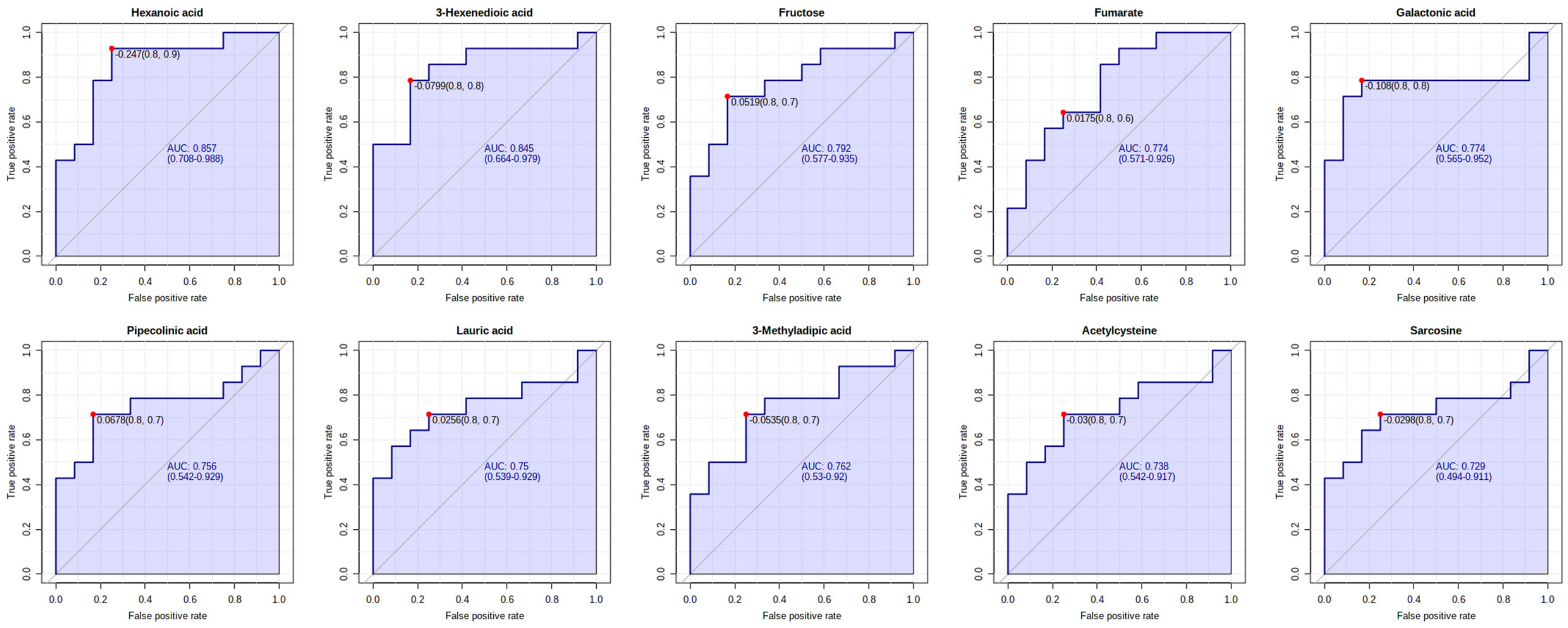

Figure S2. Univariate ROC analysis of 10 candidate metabolites with $p < 0.05$ and FC > 2.0 between groups. Results show AUC with 95% CI in addition to sensitivity and specificity estimates.
